# Identification of diagnostic markers related to oxidative stress and inflammatory response in diabetic kidney disease by machine learning algorithms: Evidence from human transcriptomic data and mouse experiments

**DOI:** 10.3389/fendo.2023.1134325

**Published:** 2023-03-07

**Authors:** Ming Zhong, Enyi Zhu, Na Li, Lian Gong, Hai Xu, Yong Zhong, Kai Gong, Shan Jiang, Xiaohua Wang, Lingyan Fei, Chun Tang, Yan Lei, Zhongli Wang, Zhihua Zheng

**Affiliations:** ^1^ Department of Nephrology, Center of Kidney and Urology, the Seventh Affiliated Hospital, Sun Yat-sen University, Shenzhen, China; ^2^ Edmond H. Fischer Translational Medical Research Laboratory, Scientific Research Center, The Seventh Affiliated Hospital, Sun Yat -Sen University, Shenzhen, China; ^3^ Department of Oncology, the Third Xiangya Hospital, Central South University, Changsha, China; ^4^ Division of Endocrinology and Rheumatology, Huangpi People’s Hospital, the Third Affiliated Hospital of Jianghan University, Wuhan, China; ^5^ Department of Clinical Medicine, Hubei Enshi College, Enshi, China; ^6^ Department of Clinical Medicine, Xiangnan University, Chenzhou, China; ^7^ Department of Internal Medicine and Geriatrics, Zhongnan Hospital, Wuhan University School of Medicine, Wuhan, China

**Keywords:** diabetic kidney disease, biomarker, diagnostic model, immune, bioinformatic analysis

## Abstract

**Introduction:**

Diabetic kidney disease (DKD) is a long-term complication of diabetes and causes renal microvascular disease. It is also one of the main causes of end-stage renal disease (ESRD), which has a complex pathophysiological process. Timely prevention and treatment are of great significance for delaying DKD. This study aimed to use bioinformatics analysis to find key diagnostic markers that could be possible therapeutic targets for DKD.

**Methods:**

We downloaded DKD datasets from the Gene Expression Omnibus (GEO) database. Overexpression enrichment analysis (ORA) was used to explore the underlying biological processes in DKD. Algorithms such as WGCNA, LASSO, RF, and SVM_RFE were used to screen DKD diagnostic markers. The reliability and practicability of the the diagnostic model were evaluated by the calibration curve, ROC curve, and DCA curve. GSEA analysis and correlation analysis were used to explore the biological processes and significance of candidate markers. Finally, we constructed a mouse model of DKD and diabetes mellitus (DM), and we further verified the reliability of the markers through experiments such as PCR, immunohistochemistry, renal pathological staining, and ELISA.

**Results:**

Biological processes, such as immune activation, T-cell activation, and cell adhesion were found to be enriched in DKD. Based on differentially expressed oxidative stress and inflammatory response-related genes (DEOIGs), we divided DKD patients into C1 and C2 subtypes. Four potential diagnostic markers for DKD, including tenascin C, peroxidasin, tissue inhibitor metalloproteinases 1, and tropomyosin (TNC, PXDN, TIMP1, and TPM1, respectively) were identified using multiple bioinformatics analyses. Further enrichment analysis found that four diagnostic markers were closely related to various immune cells and played an important role in the immune microenvironment of DKD. In addition, the results of the mouse experiment were consistent with the bioinformatics analysis, further confirming the reliability of the four markers.

**Conclusion:**

In conclusion, we identified four reliable and potential diagnostic markers through a comprehensive and systematic bioinformatics analysis and experimental validation, which could serve as potential therapeutic targets for DKD. We performed a preliminary examination of the biological processes involved in DKD pathogenesis and provide a novel idea for DKD diagnosis and treatment.

## Introduction

Diabetes kidney disease (DKD) is a chronic kidney disease caused by diabetes. About 40% of type 2 diabetes patients and 30% of type 1 diabetes patients present with DKD (1, 2). With the increasing prevalence of diabetes, the number of DKD patients has also increased (3, 4). DKD patients present different forms of kidney damage, which is characterized by continuous increase of albuminuria excretion and/or a progressive decrease in glomerular filtration rate (GFR), eventually developing into end-stage renal disease (ESRD) (5). DKD is the main cause of ESRD, and about 30% to 50% of worldwide ESRD is caused by DKD (6). Therefore, it is urgent to explore early effective diagnosis and intervention targets for exploring new diagnosis and treatment strategies to improve the clinical DKD outcome.

The pathogenesis of DKD is complex and multifactorial. Generally, DKD is mainly caused by hemodynamic changes and metabolic disturbances (7). These changes subsequently lead to activation of the renin-angiotensin-aldosterone system (RAAS) (8), increases in metabolites and pro-inflammatory factors, and dysregulation of many intracellular signaling cascades associated with oxidative stress (9–11). In the state of diabetes, on one hand, the self-oxidation of glucose causes mitochondrial overload and excessive production of reactive oxygen species (ROS). On the other hand, the body’s antioxidant capacity decreases, and the amount of intracellular antioxidant (nicotinamide adenine dinucleotide phosphate [NADPH]) is insufficient (12), resulting in an imbalance between oxidants and antioxidants. In addition, oxidative stress is also closely related to inflammatory cells, which often coexist and activate each other. Excessive oxidative stress and inflammatory responses lead to damage to the renal interstitium, glomeruli, and renal podocytes, thereby impairing renal function. Therefore, finding diagnostic and therapeutic targets for oxidative stress and inflammatory response is expected to block the process of renal injury in DKD and restore renal function.

With the popularization of gene chips and high-throughput sequencing, many disease databases have gradually been improved, and more and more effective data can be used to reveal the pathogenesis of diseases and new therapeutic targets. For example, Ma used a bioinformatic approach to analyze gene expression profiles and underlying functional networks in cardiac tissue from patients with dilated cardiomyopathy (13). Huang analyzed the correlation of serum 25-hydroxyvitamin D levels in the progression of proteinuria in DKD and its underlying mechanisms (14). Yang explored seven immune-related genes that can predict the progression of atherosclerotic plaque based on machine learning (15). However, existing studies have some deficits, such as analysis based on a single dataset, limited number of patients, and no multi-faceted validation of bioinformatics methods, which affects prediction capability or reliability. This study integrated DKD datasets from different sources, used a variety of biological information methods to screen diagnostic markers related to oxidative stress and inflammatory response in DKD, and thoroughly examined the biological functions and potential mechanisms of diagnostic markers. This discovery may provide a promising direction for clarifying the diagnosis and pathogenesis of DKD.

## Materials and methods

### Data sources and processing

DKD gene expression profiling data were downloaded from the Gene Expression Omnibus (GEO) database (https://www.ncbi.nlm.nih.gov/geo/), including seven datasets, GSE111154, GSE142025, GSE162830, GSE163603, GSE96804, GSE1009, and GSE30122. **Table 1** presents more details concerning the above datasets. Excluding the samples irrelevant to this study, 214 samples were finally obtained, including 101 normal samples and 113 DKD samples. The “sva” R package was applied for removing batch effects from different datasets (16). A Principal component analysis (PCA) was utilized to assess the effect of batch effect removal and visualize the distribution of DKD and normal patient samples. Subsequently, we obtained 458 oxidative stress-related genes from the Gene Ontology (GO) knowledgebase (http://geneontology.org/) and 200 inflammatory response related genes from MsigDB (HALLMARK_INFLAMMATORY_RESPONSE) (http://www.broad.mit.edu/gsea/msigdb/) as shown in **Supplementary Table 1**.

**Table 1:** Details of the datasets included in this study.

Datasets	Platforms	Organism	DKD	Normal	References	Status
GSE111154	GPL17586	Homo sapiens	4	4	PMID: 30253844	Public on Jul 03, 2018
GSE142025	GPL20301	Homo sapiens	27	9	PMID: 32086290	Public on Dec 14, 2019
GSE162830	GPL20301	Homo sapiens	10	9	PMID: 33537765	Public on Dec 09, 2020
GSE163603	GPL16791	Homo sapiens	9	6	PMID: 35675394	Public on Dec 22, 2020
GSE96804	GPL17586	Homo sapiens	41	20	PMID: 29242313	Public on Jul 31, 2018
GSE1009	GPL8300	Homo sapiens	3	3	PMID: 15042541	Public on Apr 01, 2004
GSE30122	GPL571	Homo sapiens	19	50	PMID: 21752957	Public on Aug 03, 2011

### Identification of DEGs and functional enrichment analysis

The “limma” R package was used for differential analysis (|log2FC|>0.5, padj < 0.05) (17). The”ggplot2” R package was applied for drawing volcano plots showing the distribution of differentially expressed genes (DEGs). The up- and down-regulated genes were analyzed using an Over-Representation Analysis (ORA). The R package “msigdbr” was used to provide a reference gene set. The enrichment analysis results from the “C2,” “C5,” and “H” gene sets were selected for visual display.

### Consensus clustering analysis of DEOIGs

The R package “ConsensusClusterPlus” was used for consensus unsupervised clustering analysis (18), and separated patients into different molecular subtypes based on the expression levels of differentially expressed oxidative stress- and inflammatory response-related genes (DEOIGs). A consensus matrix plot, consensus cumulative distribution function (CDF) plot, relative alterations in area under the CDF curve, and tracking plot to find the optimal number of clusters were used. The “clusterProfiler” R package was used for performing gene set enrichment analysis (GSEA) (19). The single sample gene set enrichment analysis (ssGSEA) analysis was utilized to quantify pathways related to DKD (20).

### Weighted gene co-expression network analysis

Based on the expression similarity of 113 DKD samples, genes were divided into different modules using the weighted correlation network analysis (WGCNA) method (21). According to the importance assessment of genes in the module and the correlation analysis between modules and subtypes, a module highly related to DKD was found, and the genes in this module were used for subsequent research.

### Screening and validation of diagnostic markers for DKD

The module genes obtained from WGCNA analysis were uploaded to the String database (https://cn.string-db.org/) for protein interaction analysis, and the protein–protein interaction (PPI) network file was exported for further analysis based on the Cytoscape 3.9.0 software. The CytoHubba plug-in of the Cytoscape 3.9.0 software was applied for screening core genes. The plug-in utilized 12 algorithms, including MCC, dmnc, MNC, degree, EPC, bottleneck, eccentricity, closeness, radiology, betweenness, stress, and clustering efficiency to score the genes and screen the genes that satisfied the 12 algorithms as candidate genes. Next, we utilized the Least Absolute Shrinkage and Selection Operator (LASSO) logistic regression (analyzed by the “glmnet” R package (22)), Random Forest (RF) as analyzed by the “randomForest” R package, and Support Vector Machine_Recursive Feature Elimination (SVM_RFE)) (23) to screen candidate genes, and the overlapping genes of the three algorithms were regarded as diagnostic markers. The expression data and clinical information data, such as creatinine and GFR of DKD were downloaded from the Nephroseq v5 online database (http://v5.nephroseq.org/) to verify the selected diagnostic markers. A receiver operating characteristic (ROC) curve was employed for evaluating the diagnostic efficacy of diagnostic markers (24).

### Establishment and validation of a nomogram scoring system

The “RMS” R package was used to develop a diagnostic model of DKD based on diagnostic markers. Using a nomogram scoring system, each variable was assigned a score, and the scores for all variables were then summed to obtain a total score for each sample. The calibration curves were used to assess the accuracy of the nomogram, and the Decision Curve Analysis (DCA) was chosen for evaluating the clinical utility of the nomogram (25).

### GSEA enrichment analysis of biological functions and pathways of diagnostic markers

The ssGSEA analysis was applied for quantifying 28 immune-related gene sets. The samples were divided into high and low expression groups according to the gene expression of each diagnostic marker, and a GSEA analysis was used to explore the biological functions and pathways associated with each gene. The “h.all.v7.5.1.symbols.gmt” and “c5.all.v7.5.1.symbols.gmt” were used as reference genomes.

### Animal experiments

Twenty-four male BKS-DB mice (Strain NO. T002407, 12 each of 6 weeks old and 12 weeks old) and twenty-four age-matched non-diabetic mice were purchased from GemPharmatech (Guangdong, China). Blood and urine samples were collected, and the mice were then sacrificed to obtain kidney samples, some of which were partially stored in 4% paraformaldehyde, and the rest were immediately stored at –80°C for subsequent studies. All animal experiments were approved by the Ethics Committee of Sun Yat-sen University (Approval No. SYSU-IACUC-2022-001575), and the entire experimental procedure was carried out in strict compliance with the Guide for the Care and Use of Laboratory Animals.

Blood was drawn *via* the tail vein and blood glucose levels were measured using an ACCU-CHEK^®^ Performa glucometer (Roche, Manheim, Germany). Blood urea nitrogen (BUN), serum creatinine (Scr), glycosylated hemoglobin (HbA1c), and urine creatinine were detected using an automatic biochemical analyzer (Chemray 800, Shenzhen, China). Urine albumin was measured using a turbidimetric inhibition immunoassay (Nanjing Jiancheng Bioengineering Institute, Nanjing, China, No. E038-1-1). Urinary albumin-to-creatinine ratio (UACR) was calculated as urine albumin/urine creatinine (μg/mg). Mouse kidney tissue fixed in 4% paraformaldehyde was rinsed, dehydrated, routinely paraffin embedded, sectioned (5 µm), and stained by Hematoxylin-eosin staining (H&E), Periodic Acid-Schiff stain (PAS), and Masson. Meanwhile, mouse kidney sections were deparaffinized, hydrated, and incubated in 10 mM sodium citrate buffer at 98°C for 20 min for antigen retrieval. The sections were incubated with primary antibody against TNC (Cat:67710-1-Ig, Proteintech, USA), PXDN (Cat: FNab10858, FineTest, China), TPM1(Cat: A1157, Abclonal, China), and TIMP1 (Cat: 106164-T08, Sinobiological, China) overnight at 4°C. Then, the sections were incubated with the secondary antibody for 1 h at room temperature. The sections were then incubated with 3,3’-diaminobenzidine for 20 min at room temperature. Stained sections were visualized and imaged with a light microscope (Olympus, Tokyo, Japan). Next, a double-sandwich enzyme-linked immunosorbent assays (ELISA) for mouse TNC and TIMP1 (Elabscience, Wuhan, China) and PXDN (SAB, Maryland, USA) were performed to determine their protein concentrations. We also performed quantitative real-time polymerase chain reaction (qRT-PCR) according to the manufacturer’s instructions (ACCURATE BIOLOGY, Changsha, China). The relative quantification of mRNA levels was calculated based on the 2−ΔΔCt method, and actin beta (ACTB) was used as an internal control. The PCR primer sequences are shown in **Supplementary Table 2**.

### Statistical analyses

The Unpaired t-Test and Wilcoxon Rank-Sum Test were used to analyze the differences between the two groups. The differences among multiple groups were analyzed by the Kruskal-Wallis test. Pearson or Spearman correlation test was used to analyze the correlation between variables. R software (version 4.0.3) and Adobe Illustrator (version 25.0) was utilized for statistical analysis and drawing. P value < 0.05 was considered statistically significant.

## Results

### Data processing

**Figure 1:**
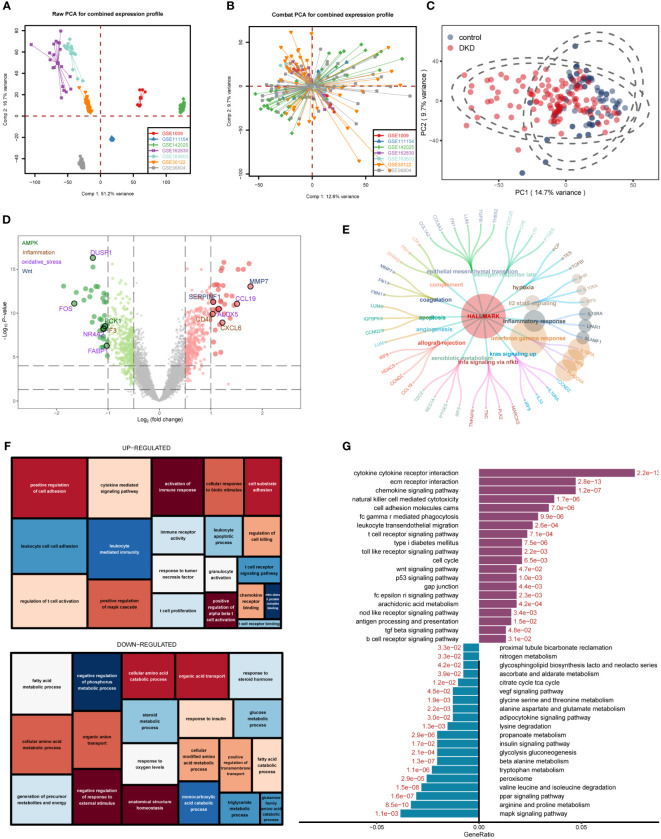
Differentially expressed gene (DEG) identification of diabetic kidney disease (DKD) and enrichment analysis. **(A, B)** Principal component analysis (PCA) showing the expression distribution of seven DKD datasets before **(A)** and after **(B)** batch effect removal. **(C)** PCA analysis revealed significant differences in transcriptome levels between DKD samples and normal samples. **(D)** Volcano plot of differentially expressed genes (DEGs) between DKD samples and normal samples. The DEGs in the pathways related to the occurrence and development of DKD reported in the literature are displayed in different colors. Hallmark gene sets **(E)**, gene ontology (GO) **(F)**, KEGG **(G)** enrichment landscape of the reference gene set.

The workflow of this study was shown in the flowchart (**Supplementary Figure 1**). We downloaded seven datasets from the GEO database with a total of 214 samples and used the “ComBat” function of the “sva” R package to remove batch effects of data from different sources. The PCA chart showed the data distribution before and after (**Figures 1A, B**, respectively) removing the batch effect, and the results indicated that the batch effect had been effectively corrected. After the data were merged, the DKD and normal samples could be accurately distinguished (**Figure 1C**). Using the “limma” R package for differential analysis, we identified a total of 772 DEGs of which 381 and 391 were up- and down-regulated, respectively, as shown in the volcano map (**Figure 1D**). Next, we performed an ORA enrichment analysis on the resulting differential genes. It can be seen from the circle network diagram that these genes were enriched in pathways, such as “INFLAMMATORY_RESPONSE,” “EPITHELIAL_MESENCHYMAL_TRANSITION,” “APOPTOSIS,” and “TNFA_SIGNALING_VIA_NFKB” (**Figure 1E**). The TreeMap revealed that up-regulated genes were mainly involved in biological processes, such as immune activation, T-cell activation, and cell adhesion, while down-regulated genes were mainly enriched in biological functions related to metabolic regulation (**Figure 1F**). These findings were correspondingly verified by Kyoto Encyclopedia of Genes and Genomes (KEGG) pathway enrichment analysis (**Figure 1G**).

### Identification of distinct subgroups in DKD

**Figure 2:**
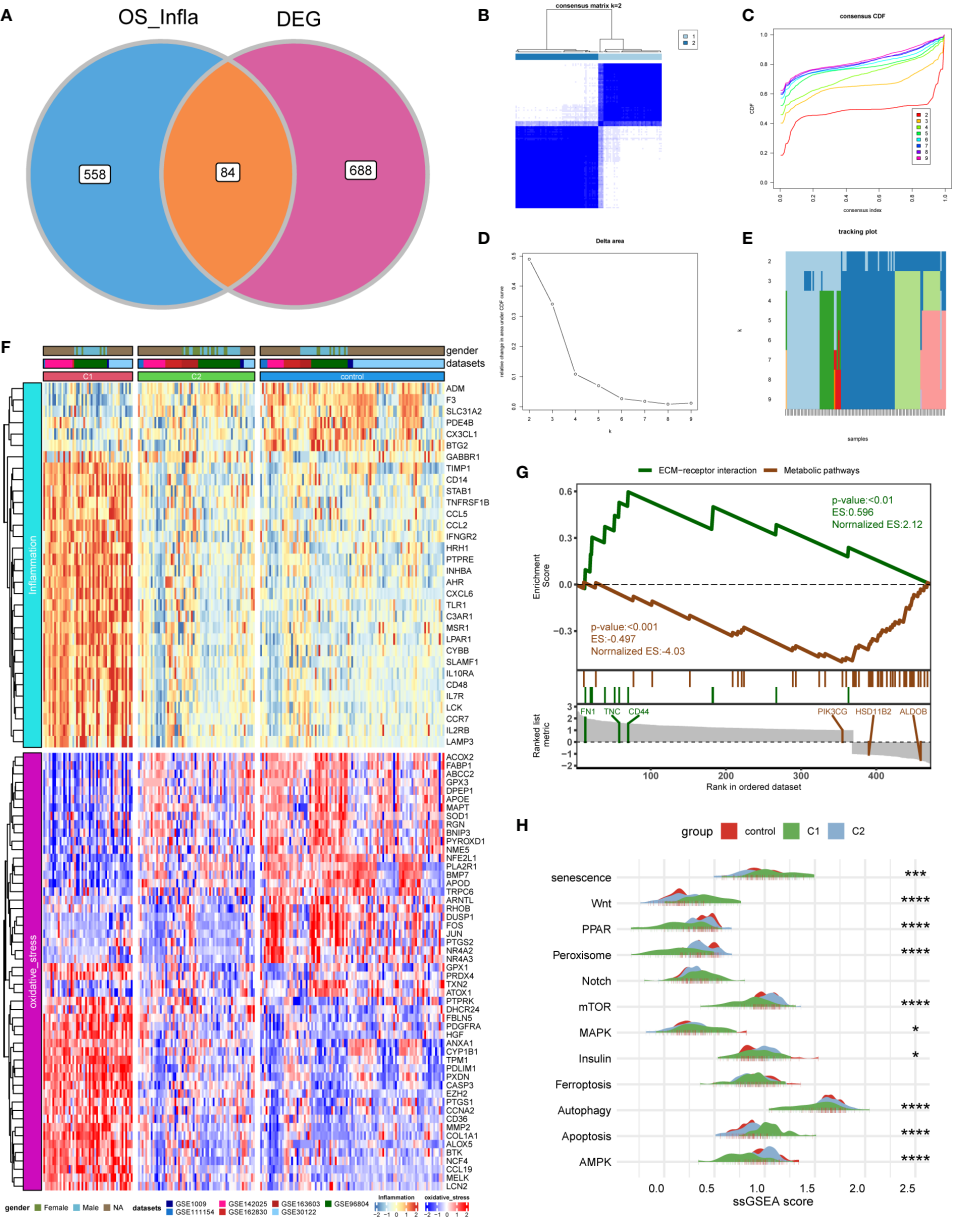
Identification of DKD subtypes. **(A)** Venn diagram showing the intersection of oxidative stress and inflammation-related genes (OS_Infla) and DEGs. **(B)** Consensus matrix when k was 2. **(C)** Consensus distribution function (CDF) when K was between 2 and 9. **(D, E)** Relative changes in the area under the CDF curve for k = 2 to 9. **(F)** Expression heatmap of genes related to oxidative stress and inflammatory response among C1 and C2 subtypes and normal samples. **(G)** A gene set enrichment analysis (GSEA) of the status of biological pathways in two DKD subtypes. **(H)** The mountain graph showing the differences in DKD characteristic pathway scores among C1 subtype, C2 subtype, and normal samples. (Kruskal–Wallis test, ****P < 0.001, ***P < 0.005, *P < 0.05).

First, we intersected oxidative stress and inflammatory response-related genes (OS_Infla) with previously obtained DEGs and obtained 84 DEOIGs (**Figure 2A**). Next, we used the R package “ConsensusClusterPlus” to classify DKD patients into different subgroups based on these 84 DEOIGs. When the consensus matrix k value was 2, the crossover among DKD samples was the smallest, which met the selection standards (**Figures 2B-E**). Consequently, 113 DKD samples were divided into two distinct clusters, DKD subtypes 1 and 2 (C1 and C2, respectively). The heatmap showed that most DEOIGs were up-regulated in the C1 subtype, while they were down-regulated in the C2 subtype and normal samples (**Figure 2F**). The GSEA enrichment analysis indicated that ECM-receptor interactions were enriched in the C1 subtype, while metabolic pathways were enriched in the C2 subtype (**Figure 2G**). We quantified the ssGSEA enrichment scores of different immune cell subgroup to be used for investigating the relationship between DKD subtypes and immune cells. The results indicated that the C1 subtype was enriched in more immune-related cells, such as regulatory T-cells, macrophages, activated B-cells, and plasmacytoid dendritic cells (**Supplementary Figure 2**). We then found the pathways that have been reported to be closely related to DKD in recent years by consulting the literature and quantifying the resulting pathways using a ssGSEA analysis. The mountain map showed the pathway ssGSEA score of the two subtypes and normal samples, which revealed that the Wnt, Notch, and apoptosis pathway were high in C1 subtype, and peroxiding proliferator-activated receptor (PPAR), peroxisome, mammalian target of rapamycin (mTOR), autophagy, AMPK, and other pathways were lower in the C1 subtype (**Figure 2H**).

### Construction of WGCNA and identification of key modules

**Figure 3:**
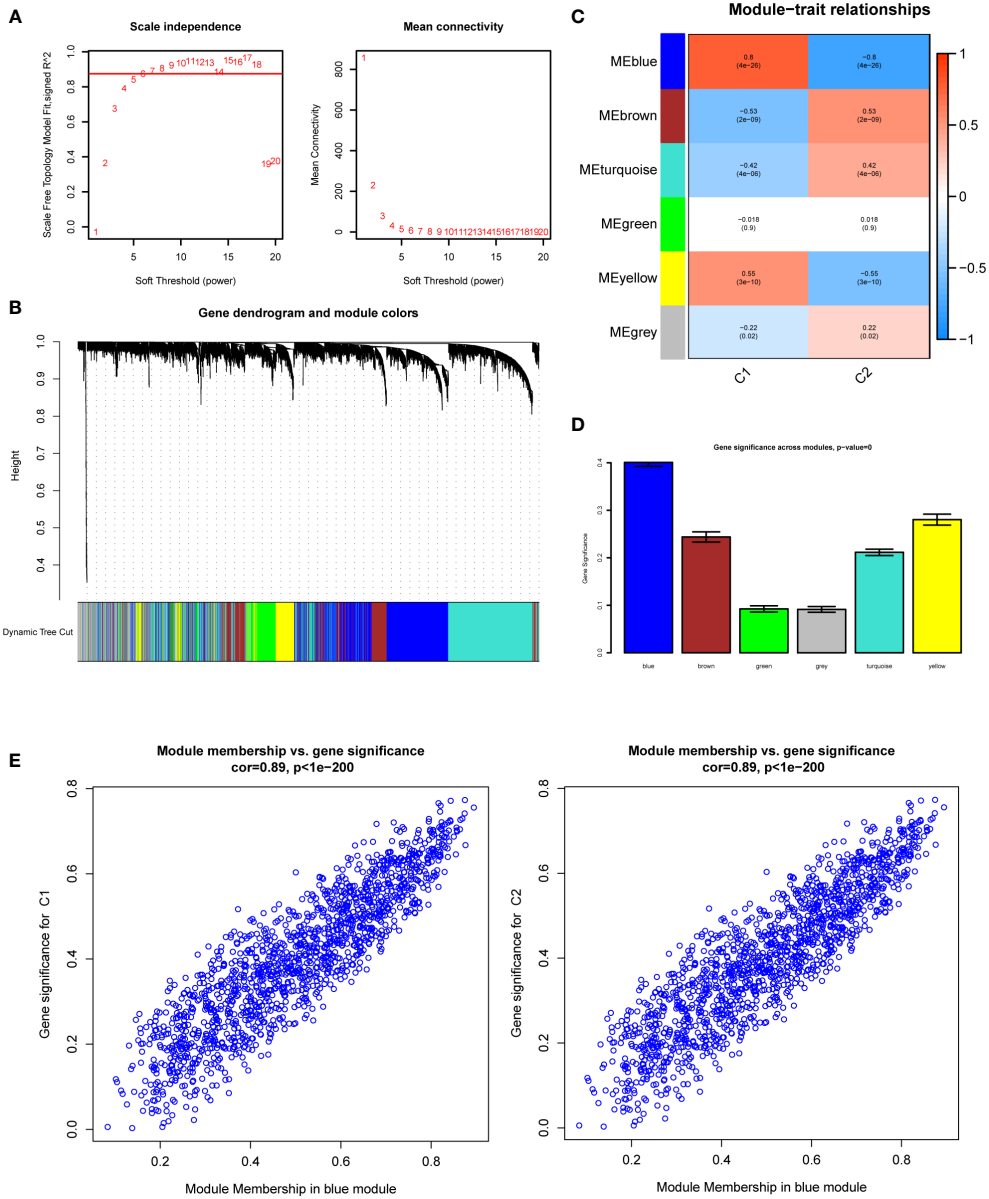
Weighted Gene Co-Expression Network Analysis (WGCNA). **(A)** Scale-free fit index and network connectivity under different soft thresholds. **(B)** The gene hierarchical clustering dendrogram. The modules corresponding to the branches are marked with color represented by the color band under the tree. **(C)** Heatmap of the correlation between gene modules and DKD subtypes. **(D)** Absolute value comparison of the correlation between genes within each module and DKD subtypes. **(E)** Scatter plot of module eigengenes in blue module.

We used 113 DKD samples from seven different datasets to screen the top 5000 genes using the median absolute deviation for the WGCNA analysis. Subsequently, we evaluated the scale-free fitting index and average connectivity of various soft threshold powers on the basis of the scale-free R2. Our study selected the soft-threshold power of β = 6 and scale-free R2 = 0.8744133 to construct a standard scale-free network with the Pick Soft Threshold function (**Figure 3A**). Ultimately, we identified six modules (**Figure 3B**). A correlation heatmap was used to explore the correlation of each module with diabetic kidney disease, and we found the MEblue module with the highest correlation with C1 and C2 subtypes (**Figure 3C**). The gene significance score was applied for analyzing the association between genes and DKD subtypes, which showed that MEblue had the highest gene significance score (**Figure 3D**). The correlation scatterplot further demonstrated that the genes in MEblue module not only strongly correlated with the MEblue module but also significantly correlated with the diabetic kidney disease subtypes (**Figure 3E**). Thus, we extracted genes in the MEblue module for subsequent analysis.

### Identification of diagnostic markers in diabetic kidney disease

**Figure 4:**
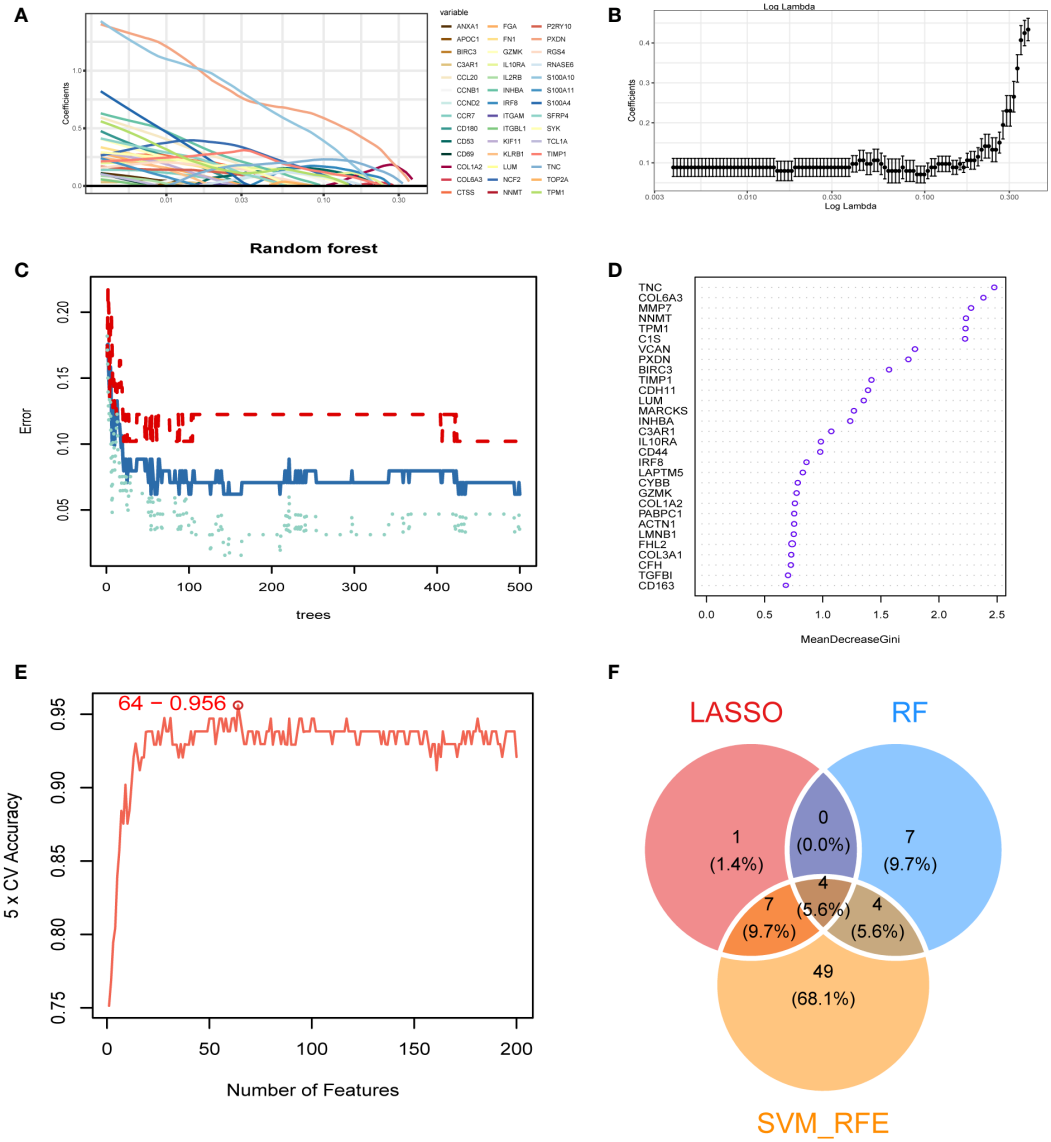
Identification of diagnostic markers. **(A, B)** A least operator shrinkage and selection operator (LASSO) logistic regression was used to screen characteristic variables. **(C)** The relationship between the number and the error of random forest. Red, green, and blue represent the error of C1 subtype, C2 subtype and all samples. **(D)** Ordination plot of gene importance scores. **(E)** The accuracy curve of characteristic variables for the first 200 genes using the support vector machine–recursive feature eliminator (SVM–RFE) algorithm. The red circle indicates the position with the highest accuracy. **(F)** Venn diagram showing the intersection feature variables filtered by the three algorithms.

We obtained 473 differential genes (|log2FC| > 1, padj < 0.05) through a differential analysis of the two subtypes in diabetic kidney disease. A Venn diagram revealed that after intersecting with the 1458 genes in the MEblue module, 347 intersecting genes were found (**Supplementary Figure 3**). The PPI network diagrams of the above-described 347 genes were constructed using the STRING online network tool, and the exported results were analyzed in Cytoscape software. The Upset plot was used to pick the intersecting genes that satisfied the 12 algorithms of the CytoHubba plugin, and finally we obtained 279 genes (**Supplementary Figure 4**). Based on these 279 genes, we further screened diagnostic markers using different bioinformatic methods. Using the LASSO regression algorithm, 12 genes were picked as potential biomarkers (**Figures 4A, B**). The random forest (RF) algorithm identified 15 candidate genes (**Figures 4C, D**). The SVM–RFE algorithm showed that when the number of eigengenes genes was 64, the accuracy was the highest up to 0.956 (**Figure 4E**). Ultimately, we obtained four genes as diagnostic markers for DKD (**Figure 4F**).

### Diagnostic value and validation of four diagnostic markers

**Figure 5:**
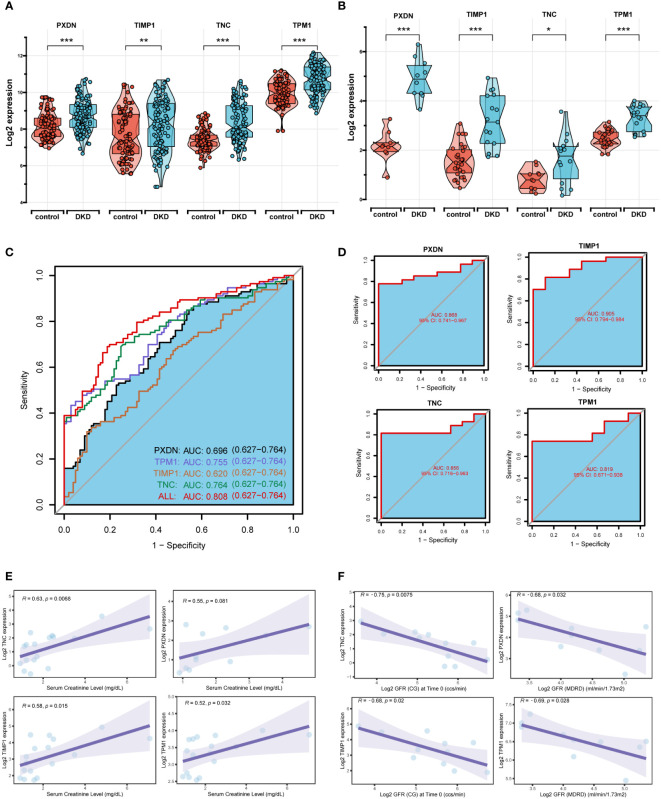
Diagnostic efficacy and external verification of diagnostic markers. **(A, B)** The 4 diagnostic markers expression in seven DKD pooled datasets **(A)** and external datasets **(B)**. **(C, D)** Receiver operating characteristic (ROC) curves assessing the diagnostic efficacy of four diagnostic markers in seven DKD combined datasets **(C)** and GSE142025 dataset **(D)**. **(E, F)** Correlation analysis of gene expression levels with creatinine **(E)** and glomerular filtration rate (GFR) **(F)**. ****P < 0.001, ***P < 0.005, **P < 0.01, *P < 0.05.

The boxplot showed the expression of the four signature genes in the seven combined GEO datasets (**Figure 5A**). It can be seen that expression of the four genes in DKD samples was higher than that in normal samples. The samples in the Nephroseq v5 online database also verified their high expression (**Figure 5B**), indicating their potential roles during the occurrence and development of DKD. In the combined GEO dataset, we found that the area under the curve (AUC) of the ROC curve was 0.808 when all four genes were fitted into one variable, which yielded a better result than when they were used alone as diagnostic variables (**Figure 5C**). We also assessed the diagnostic efficacy of these four genes in an independent patient cohort from the GSE142025 dataset. The AUC values of the ROC curves for each gene were all greater than 0.8 showing that these four genes could diagnose DKD (**Figure 5D**). Correlation analysis showed that the expression of four genes positively correlated with creatinine (**Figure 5E**) and negatively correlated with GFR (**Figure 5F**).

### Nomogram construction of DKD diagnosis model based on characteristic genes

**Figure 6:**
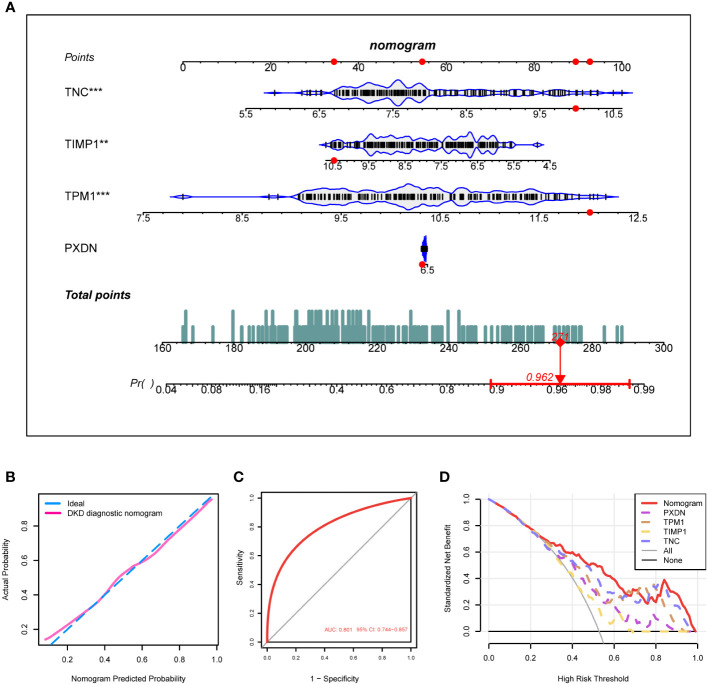
Construction of the DKD diagnostic model. **(A)** Nomogram of the DKD diagnostic model on the basis of 4 diagnostic markers. **(B, C)** The calibration **(B)** and ROC curves **(C)** were used to evaluate the diagnostic efficacy of the DKD diagnostic nomogram. **(D)** DCA illustrating the NB assessing the outcome. ****P < 0.001, ***P < 0.005, **P < 0.01, *P < 0.05.

Based on the expression of the four diagnostic markers, we constructed a diagnostic model based on logistic regression and drew a nomogram (**Figure 6A**). In the nomogram, each gene involved in the construction of the diagnostic model corresponded to a score, and their scores were added to obtain a total score, which corresponded to different diagnostic effects of DKD. The calibration curve showed that the nomogram could reliably diagnose DKD (**Figure 6B**). The ROC curve indicated that the AUC value of this model was 0.801 (**Figure 6C**). DCA results showed the net benefit (NB) evaluating the DKD patients’ outcomes through the four individual genes or a combination of them. The results illustrated that the combined nomogram model could lead to a significant increase in the NB (**Figure 6D**).

### Functional enrichment analysis of diagnostic markers

**Figure 7:**
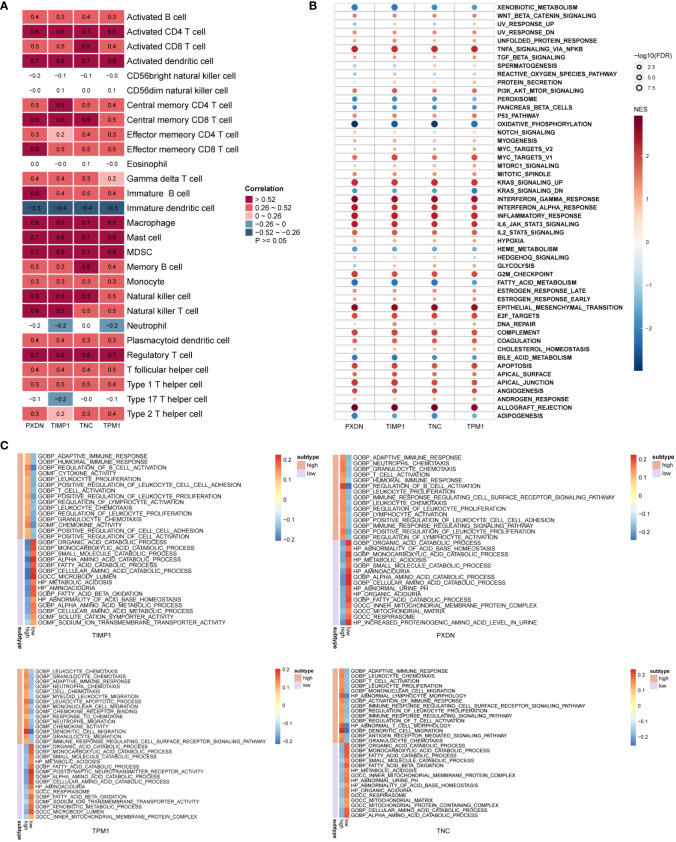
Biological function enrichment of diagnostic markers. **(A)** Heatmap of the correlation between diagnostic markers and immune cells. **(B, C)** GSEA enrichment analysis when the reference gene sets are hallmark gene sets **(B)** and ontology gene sets **(C)**.

To explore the biological processes involved in diagnostic markers, we analyzed the correlation of these four diagnostic markers with immune cells. The results indicated that they positively correlated with most immune cell infiltration (**Figure 7A**), such as activated CD4 T-cells, activated dendritic cells, regulatory T-cells, macrophages and others. Next, we divided the DKD samples into high and low expression groups based on gene expression. The differentially expressed genes in the high and low expression groups were subject to GSEA analysis to explore the possible signal pathways involved, and it was found that the pathway enrichment of the four genes was consistent. As a result, all were significantly enriched in TNFA_SIGNALING_VIA_NFKB, KRAS_SIGNALING_UP, INTERFERON_GAMMA_RESPONSE, INFLAMMATORY_RESPONSE, EPITHELIAL_MESENCHYMAL_TRANSITION (**Figure 7B**). Functional enrichment showed that the high expression groups of the four genes were all enriched in ADAPTIVE_IMMUNE_RESPONSE, T_CELL_ACTIVATION, IMMUNE_RESPONSE_REGULATING_CELL_SURFACE_RECEPTOR_SIGNALING_PA THWAY. The low expression group was enriched in biological processes, such as SMALL_MOLECULE_CAT ABOLIC_PROCESS, FATTY_ACID_CATABOLIC_PROCESS, INNER_MITOCHONDRIAL_MEMBRANE_PROTEIN_COMPLEX (**Figure 7C**).

### Validation in animal models

**Figure 8:**
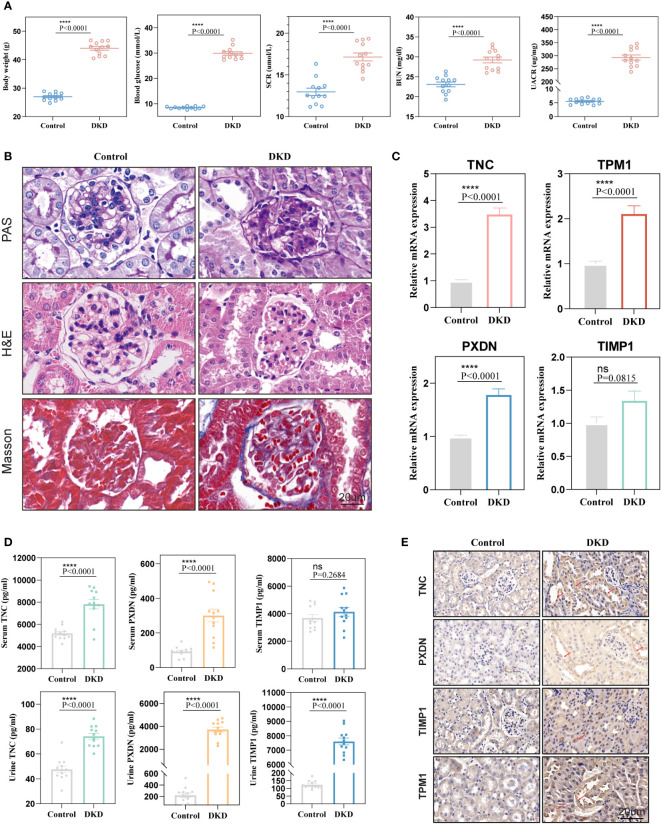
Validation of diagnostic markers in animal experiments. **(A)** The levels of body weight, blood glucose, serum creatinine, blood urea nitrogen, and urine albumin-creatinine ratio in mice. **(B)** Hematoxylin and eosin (H&E), periodic acid Schiff (PAS), Masson staining of mouse kidney. **(C)** mRNA expression levels of four diagnostic markers in kidney tissue. **(D)** Expression levels of markers in blood and urine. **(E)** Representative immunohistochemical staining of the kidneys for the four markers. ns, P < 0.05.

To further verify the diagnostic value of the four markers in the diagnosis of early DKD, we utilized 12-week-old db/db mice as a model of spontaneous DKD. We found that body weight, blood glucose, HbA1c, serum creatinine, blood urea nitrogen, and urine albumin/creatinine levels were significantly increased in DKD group mice compared with normal control mice (**Figure 8A**, **Supplementary Figure 5**). Pathological staining also showed mesangial cell proliferation, mesangial matrix expansion, and irregular thickening of glomerular and tubular basement membranes in the kidney tissue of DKD group mice (**Figure 8B**), indicating that the spontaneous DKD model had been successfully established. Next, we detected the mRNA expression levels of four biomarkers, including TNC, PXDN, TIMP1, and TPM1. The results showed that TNC, TPM1, and PXDN were significantly elevated in the mouse model. Unfortunately, TIMP1 had an upward trend and no difference between the two groups was found (**Figure 8C**). We also detected three secreted proteins among four biomarkers in mouse blood and urine. The results showed that TNC and PXDN were consistently elevated in blood and urine while TIMP1 was significantly elevated in urine but not significantly different in blood (**Figure 8D**). Correlation analysis showed that whether in blood samples or urine samples, these markers had obvious positive correlation with UACR. As for blood glucose and HbAc1, the markers were not significantly correlated with them (**Supplementary Figures 6**, **7**). Immunohistochemical results showed that the expression levels of TNC, TPM1, TIMP1, and PXDN were elevated in the DKD mouse model (**Figure 8E**). To further verify that the above changes are related to DKD rather than diabetes, our study also added two groups of 6-week-old db/db mice and normal mice. We found that body weight, blood glucose, HbA1c were significantly increased in DM mice compared with normal control mice, but there were no differences in serum creatinine, blood urea nitrogen, and urine albumin/creatinine levels between the two groups of mice (**Supplementary Figures 8A, B**). Meanwhile, no significant difference was found in renal pathological staining (**Supplementary Figure 8C**). The results of qRT-PCR showed that there was no statistical difference in the mRNA expression levels of TPM1 and TIMP1 between the two groups. The expression of TNC and PXDN increased in the DM group (**Supplementary Figure 8D**). In addition, the expression levels of three secreted proteins were detected in the blood and urine samples of mice in the DM group and 6-week-old normal mice, and we found that only TNC in the blood samples was significantly increased in the DM mice. For urine samples, there were significant differences in the elevation of TNC and TIMP1 in DM mice (**Supplementary Figure 8E**).

## Discussion

DKD is a common complication of diabetes (26) and the leading cause of ESRD, which imposes a heavy burden on people and has a noteworthy influence on health and quality of life (27). Finding and mining DKD clinical biomarkers may effectively slow down or even stop the progression of DKD. Recently, a large number of studies have made many efforts to explore new targets of DKD. Diao identified eight hub genes of DKD, such as Scd5, Coasy, and Idi1, by constructing a PPI network (28). Han used machine learning to obtain two diagnostic markers of protein kinase cAMP-dependent type II regulatory subunit beta and transforming growth factor beta 1 (PRKAR2B and TGFBI, respectively) (29) in glomerular injury in diabetic nephropathy. Wei screened and identified biomarkers in early DKD based on WGCNA, and initially explored the biological functions of candidate markers (30). However, the existing biomarkers are not enough to address the common DKD. At present, the number of samples included in most bioinformatics analysis studies is too small, and no research exploring early diagnostic markers based on the pathogenesis of DKD is available. Therefore, we still urgently need to uncover potential biomarkers with high specificity and sensitivity for pathogenesis.

In this study, we downloaded multiple DKD datasets from the GEO database, included a larger number of samples, merged the datasets by removing batch effects, and obtained differential genes between DKD patients’ kidney tissues and normal kidney tissues through differential analysis. The enrichment analysis of differential genes showed that biological processes such as immune activation, T-cell activation, and cell adhesion were enriched in DKD, which was consistent with previous reports (31, 32) in which immune regulation was found to be involved in the occurrence and progression of DKD, and many pro-inflammatory cytokines and chemokines played a vital role in the DKD pathogenesis. It was reported that oxidative stress and inflammatory response was an important pathogenesis of DKD (33, 34). For further examination of the DKD pathogenesis, we divided DKD patients into C1 and C2 subtypes based on DEOIGs. Through a GSEA analysis, it was found that extra cellular matrix (ECM)-receptor interaction was enriched in the C1 subtype with high DEOIGs expression, while metabolism-related pathways were enriched in the C2 subtype with low DEOIGs expression. As reported in related studies, ECM organization and ECM structural components could lead to accelerated extracellular matrix deposition and renal fibrosis in DKD (35), and metabolic disorders were found to play a key role in the development of DKD (36).

Machine learning is often used to find the key genes of diseases. Wang et al. found the key genes of chronic kidney disease through the WGCNA method (37). Liu et al. found the trait-related module through the WGCNA method and identified the key gene FCER1G (38). Their research revolves around the WGCNA method and the PPI interaction network to find markers and conduct experimental verification. On this basis, our research adds bioinformatics methods for screening markers, such as Lasso, RF, SVM_RFE. Ultimately, we obtained four potential DKD diagnostic markers, namely TNC, PXDN, TIMP1, and TPM1. TNC is a large hexameric extracellular matrix glycoprotein expressed in most normal adult tissues (39). TNC is significantly up-regulated in damaged and inflamed tissue (40), and it has also been reported to be independently associated with increased cardiovascular adverse events and death in patients with type 2 diabetes (41). However, few studies on the role of TNC in the progression of DKD are available. PXDN encodes a heme-containing peroxidase secreted into the extracellular matrix, which is involved in extracellular matrix formation and may play a role in the physiological and pathological fibrotic responses of the fibrotic kidney. TIMP1 belongs to the TIMP gene family, and the protein encoded by this gene family is a natural inhibitor of matrix metallopeptidase, which can regulate cell differentiation, migration, and cell death. It has been reported that plasma levels of TIMP1 are associated with early diabetic neuropathy and nephropathy in patients with type 1 diabetes (42). TPM1is a member of the highly conserved tropomyosin family, widely distributed actin-binding proteins that are involved in the contractile system of striated and smooth muscles and the cytoskeleton of non-muscle cells. No previous studies have reported the role of TPM1 in DKD pathogenesis. We found that these four genes had excellent diagnostic value in DKD (AUC > 0.8) and were positively associated with creatinine and negatively associated with GFR in DKD patients. Targeting the four genes identified by our analysis may be a promising approach for DKD treatment. Most notably, we have also developed a nomogram combining four diagnostic markers with high AUC values and good calibration that showed excellent accuracy and reliability in the diagnosis of DKD. It will hopefully be applied in the clinic and contribute to the early diagnosis of DKD. It has been reported that immune regulation correlates with the occurrence and development of DKD (31, 32). To further explore the role of these four diagnostic markers in immune regulation, we found that these four genes may be involved in TNFA_SIGNALING_VIA_NFKB,KRAS_SIGNALING_UP, INTERFERON_GAMMA_RESPONSE, INFLAMMATORY_RESPONSE and other signaling pathways through GSEA analysis thus providing a theoretical basis for our further research. It is worth noting that any bioinformatics analysis needs to be validated experimentally, so we constructed a model of spontaneous DKD and DM. Through a variety of experiments, the results showed that the mRNA and protein levels of TNC, PXDN, and TPM1 in the kidneys of the DKD model mice were consistently elevated. In the mice of DM model, their expression either had no significant difference, or the increase was not obvious. We also detected significant differences in blood and urine compared to the control group, suggesting that the three biomarkers we selected deserve further investigation. It is undeniable that the experimental results of TIMP1 are questionable. This discrepancy may be related to the type of DKD model that we constructed or to species differences. After all, our model is based on mice, and our bioinformatics analysis is based on human sample analysis. Combined with previous studies, we can see that the markers we found were elevated in DKD samples as were the previous biomarkers. In contrast, our biomarkers were significantly elevated in blood and urine with good sensitivity. In addition, the biomarkers we found are easy to detect, are fairly low-cost, and have good clinical applicability. It is worth mentioning that the spontaneous DKD model we constructed can reflect the early manifestations of DKD. The increase of markers means that DKD can be detected earlier, providing a new idea for clinical diagnosis. Our study also has some limitations in which the deeper mechanism exploration requires a large number of experiments to verify the results, which is our subsequent experimental plan in the future.

In conclusion, we identified TNC, PXDN, TIMP1, and TPM1 as potential diagnostic markers for DKD using a comprehensive and systematic bioinformatics analysis and experimental validation, established a nomogram containing these four diagnostic markers, and preliminarily explored their possible biological functions in the occurrence and development of DKD. These findings will provide a novel idea for the early diagnosis and treatment of DKD.

## Data availability statement

The datasets presented in this study can be found in online repositories. The names of the repository/repositories and accession number(s) can be found in the article/**Supplementary Material**.

## Ethics statement

All animal experiments were approved by the Ethics Committee of Sun Yat-sen University, and the entire experimental procedure was carried out in strict compliance with the Guide for the Care and Use of Laboratory Animals.

## Author contributions

MZ analyzed and interpreted the data. MZ, EZ, NL and LG wrote the manuscript. HX, YZ, KG, SJ, XW, LF, CT and YL edited the manuscript. ZZ and ZW designed and edited the manuscript. All authors contributed to the article and approved the manuscript.
