# Supplementary material for: Identification of diagnostic markers related to oxidative stress and inflammatory response in diabetic kidney disease by machine learning algorithms: Evidence from human transcriptomic data and mouse experiments
Source: Front Endocrinol (Lausanne). 2023 Mar 7;14:1134325. doi: 10.3389/fendo.2023.1134325 (PMC10028207; doi:10.3389/fendo.2023.1134325)
Supplement: Supplementary file 1 [file Table_1.docx]

**Supplementary Materials**

**Supplementary Figures**

**Supplementary Figure 1.**

**
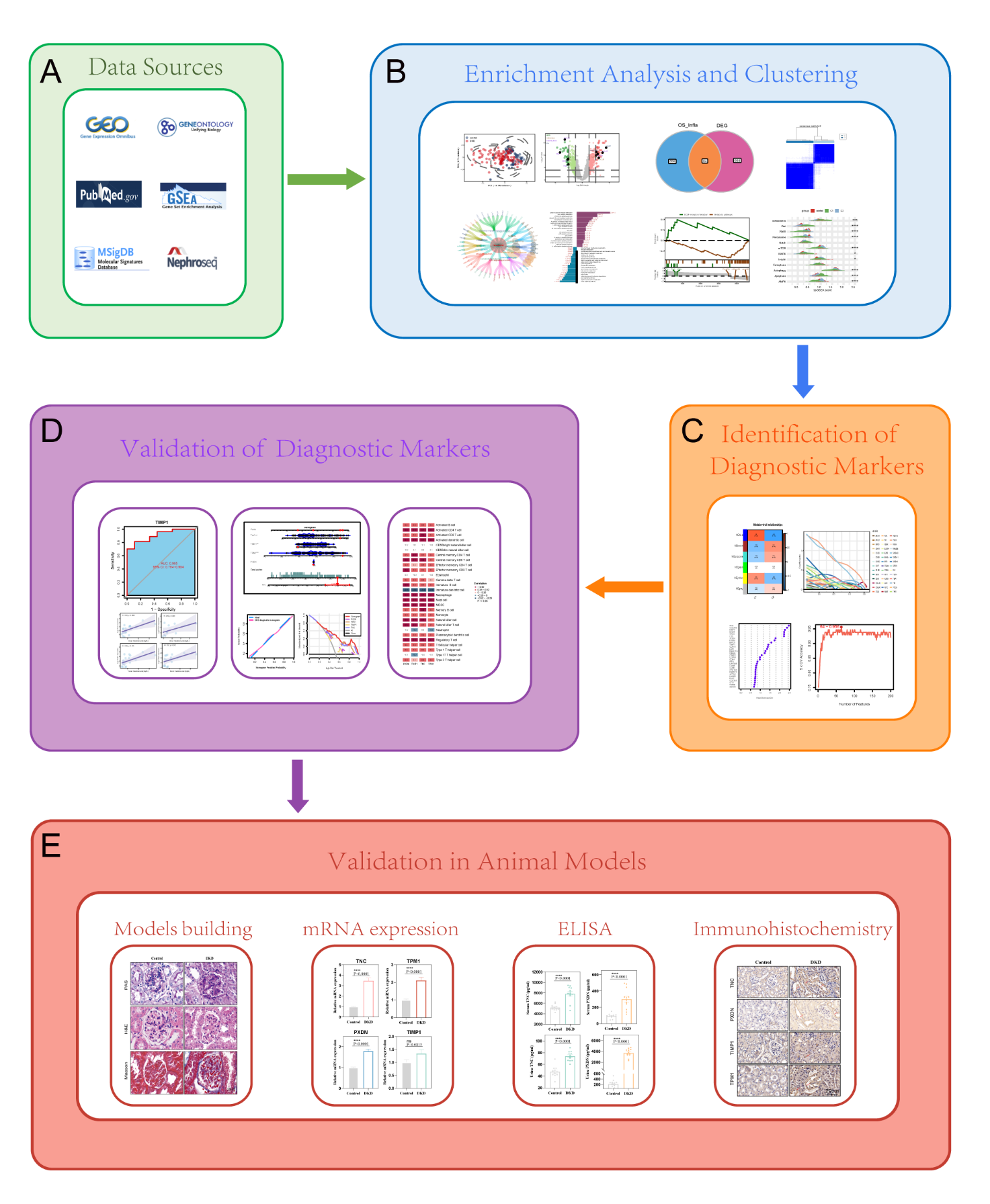
**

**Supplementary Figure 1.** Workflow of this study. (A) Analytical datasets and sources of validation datasets used in this study. (B) Identification of differential genes and analysis of biological functions and pathways. (C) Multiple bioinformatic approaches to identify diagnostic markers. (D) Validation of four diagnostic markers and construction of diagnostic models. (E) Construction of a diabetic kidney disease (DKD) mouse model and confirmation of the reliability of the four diagnostic markers.

**Supplementary Figure 2.**

**
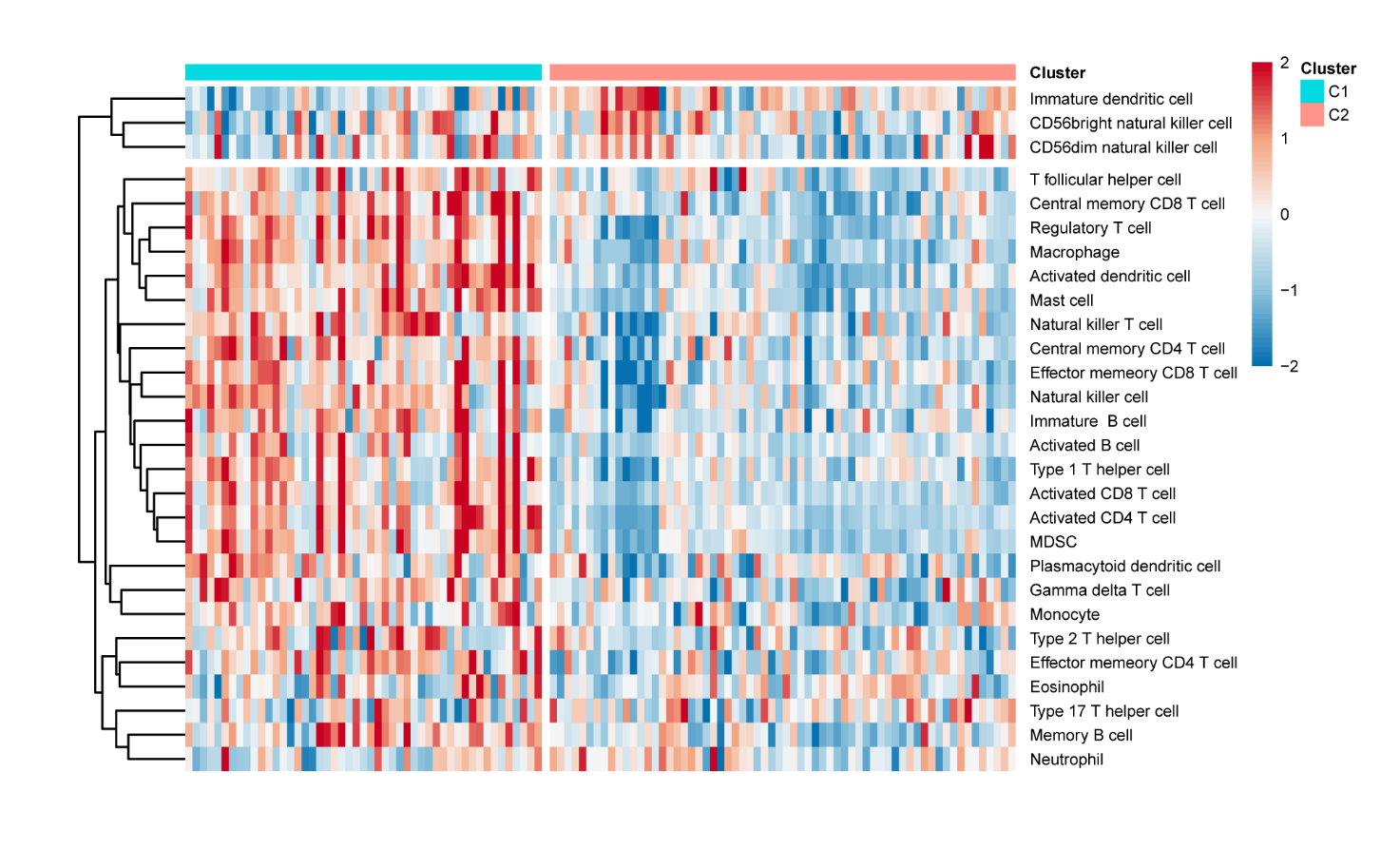
**

**Supplementary Figure 2.** The heatmap showing immune cell infiltration of DKD subtypes.

**Supplementary Figure 3.**

**
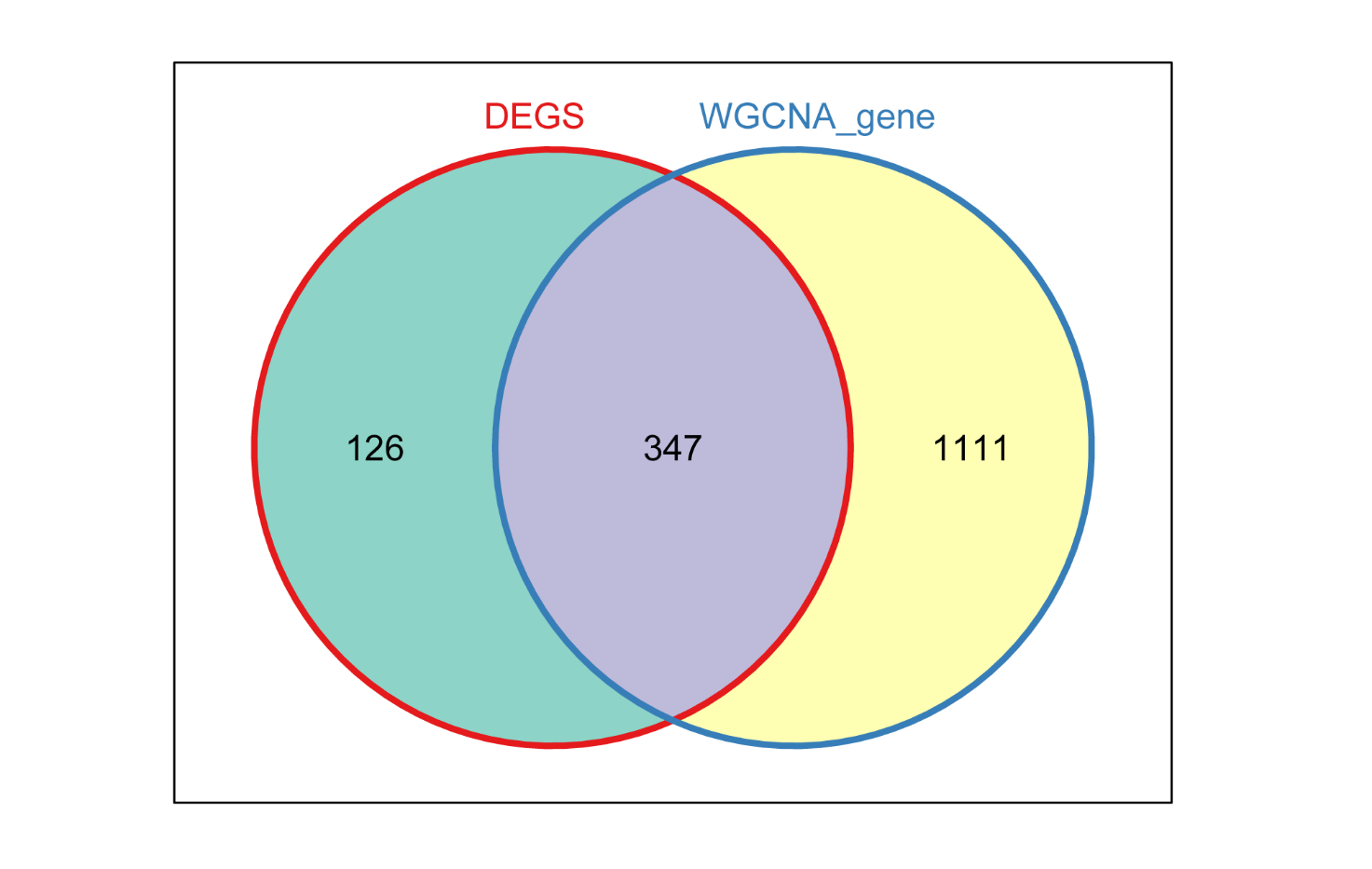
Supplementary Figure 3.** Intersection of differentially expressed genes (DEGs) of the two DKD subtypes with MEblue module genes in WGCNA.

**Supplementary Figure 4.**

**
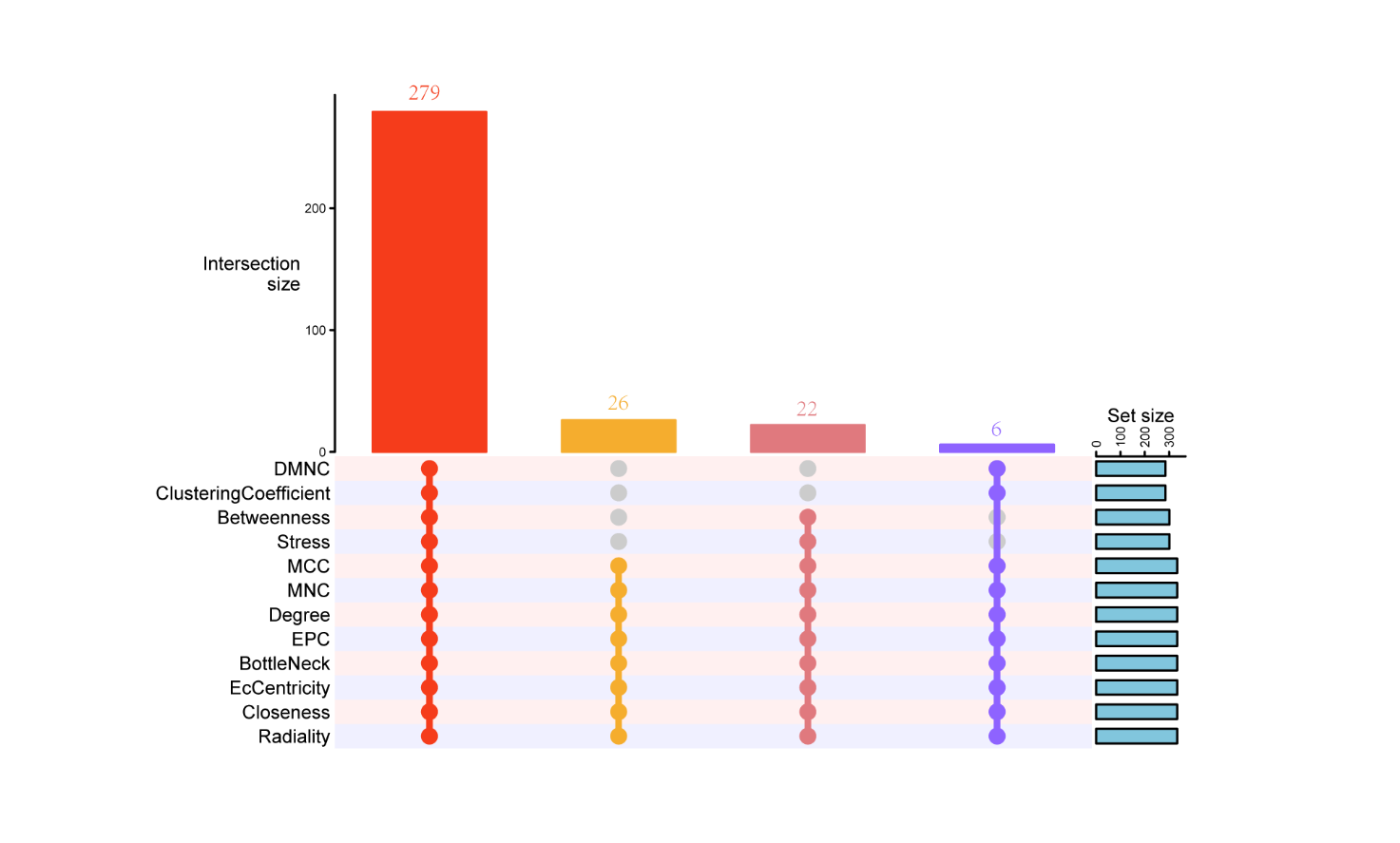
Supplementary Figure 4.** The hub genes were selected using 12 algorithms in the CytoHubba plug-in of Cytoscape software, and the genes satisfying the 12 algorithms were identified as candidate genes.

**Supplementary Figure 5.**


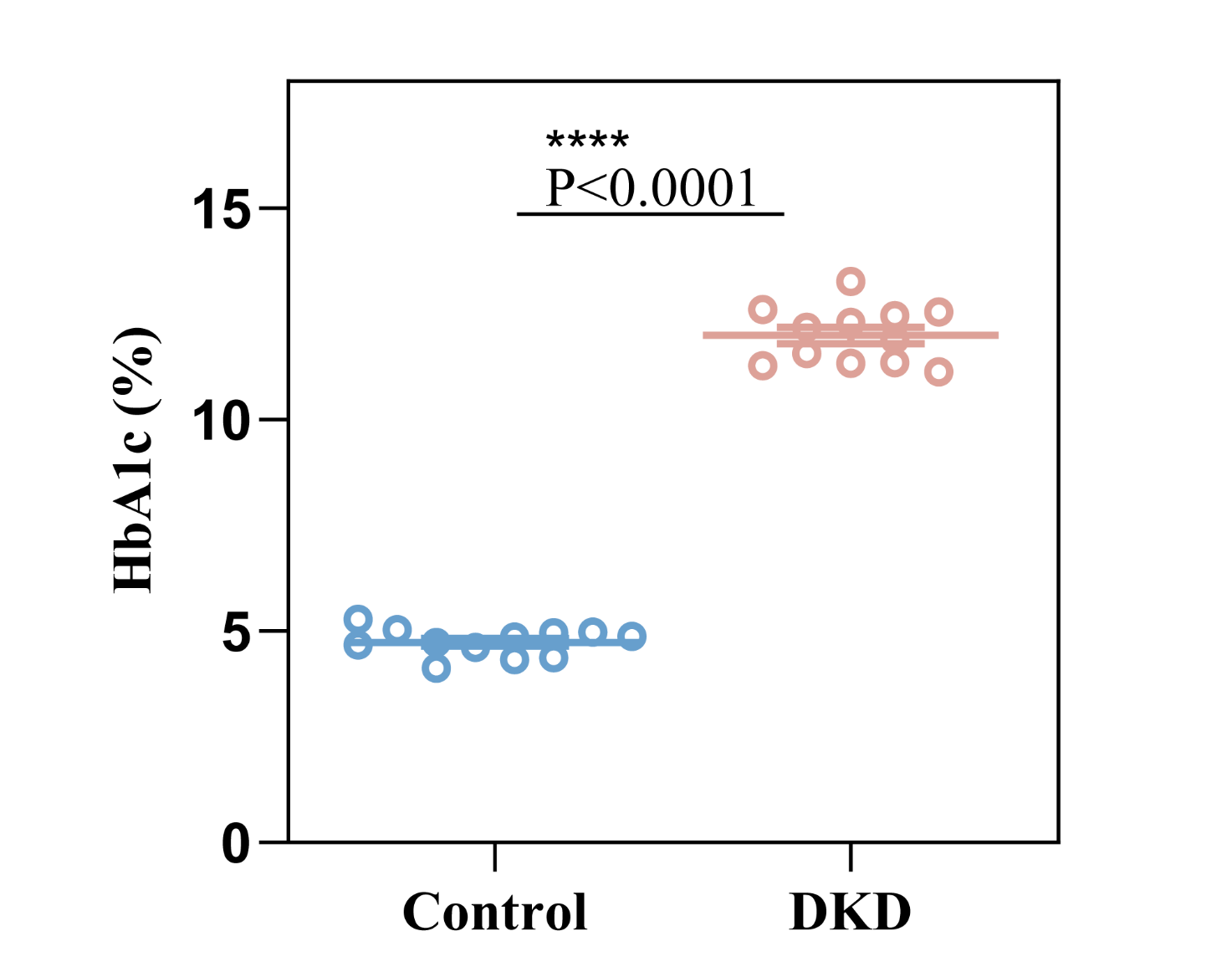


**Supplementary Figure 5.** HbA1c in control group mice and DKD group mice.

**Supplementary Figure 6.**


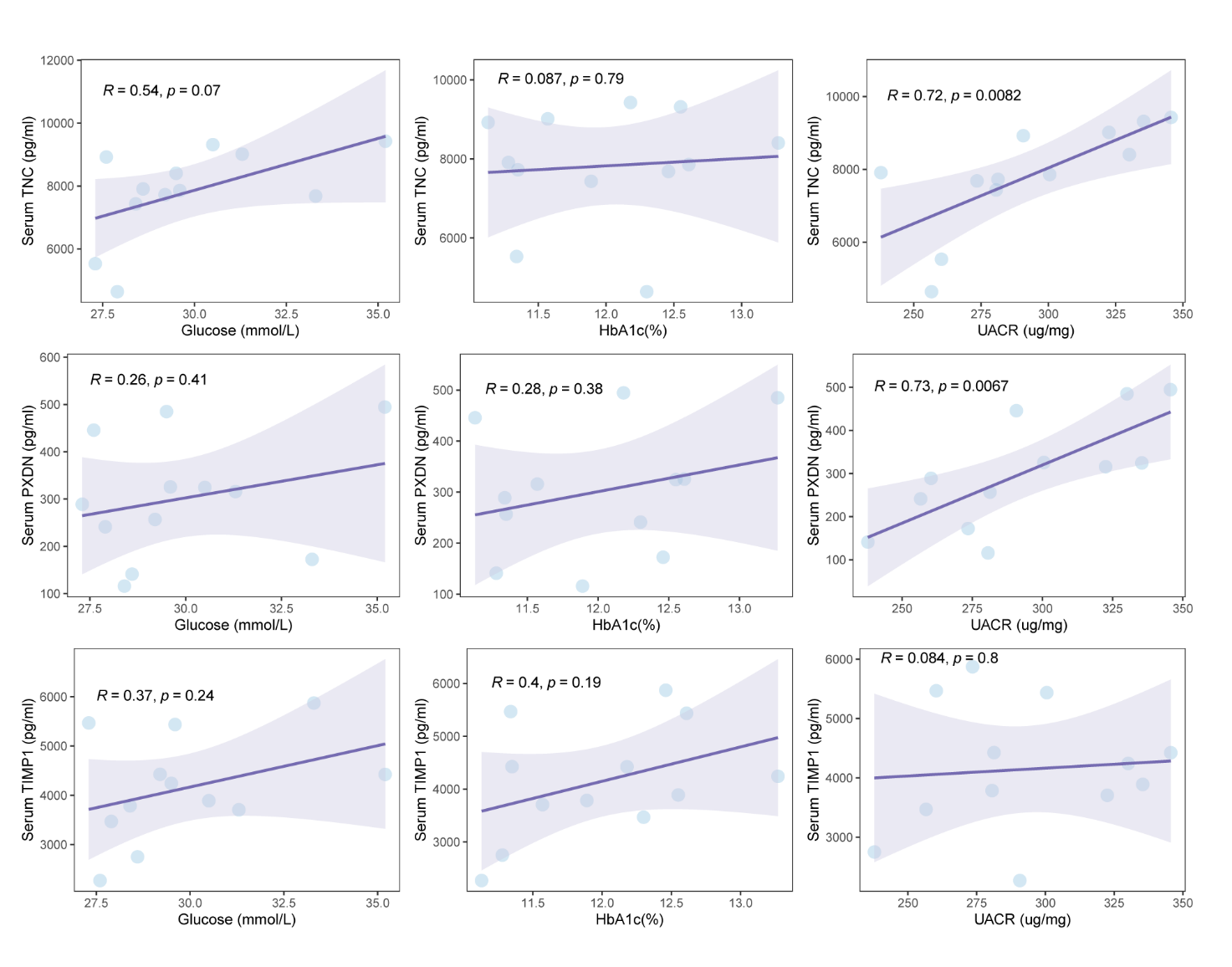


**Supplementary Figure 6.** Correlation between the expression of diagnostic markers and blood glucose, HbA1c, UACR in blood samples of DKD mice.

**Supplementary Figure 7.**


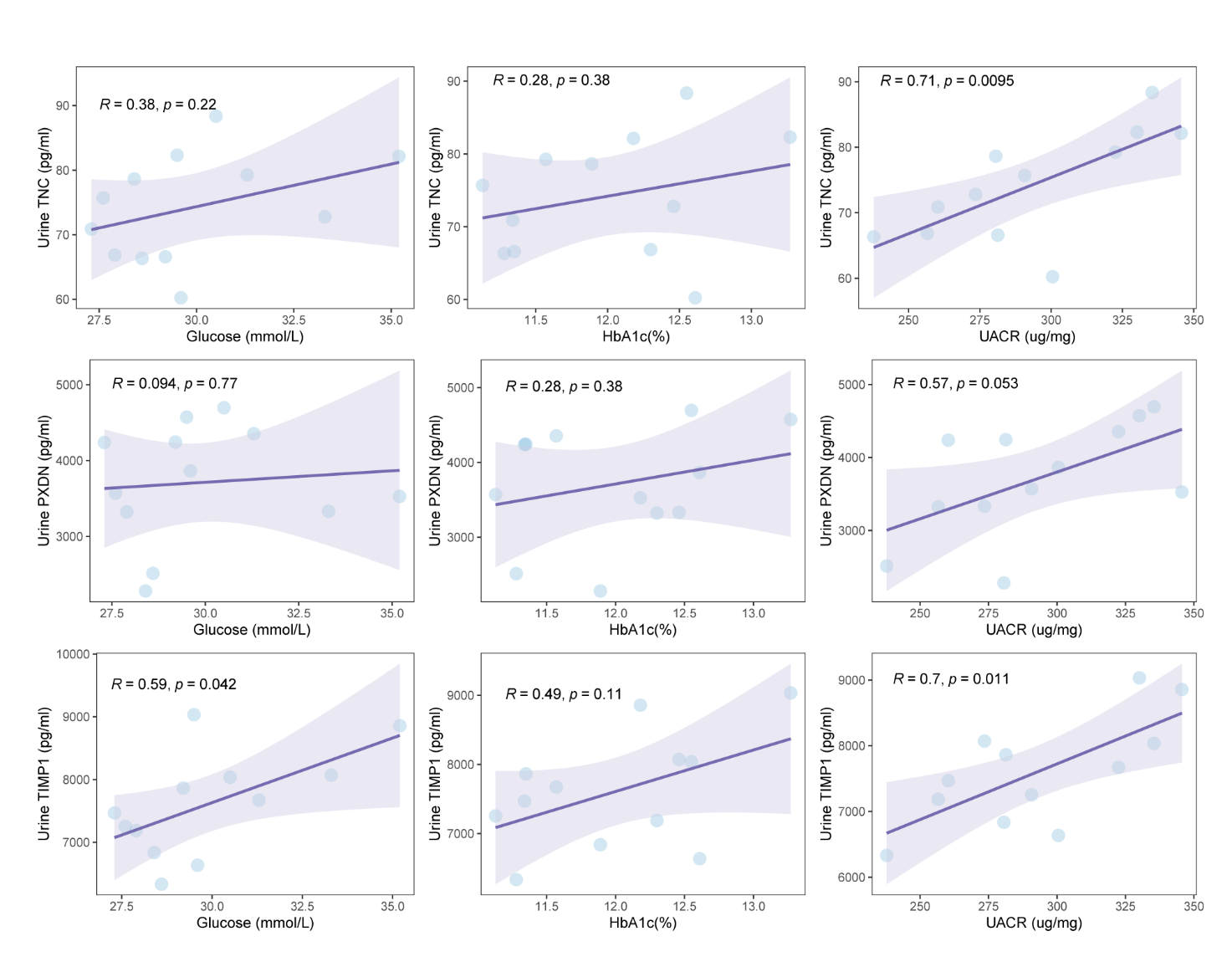


**Supplementary Figure 7.** Correlation between the expression of diagnostic markers and blood glucose, HbA1c, UACR in urine samples of DKD mice.

**Supplementary Figure 8.**


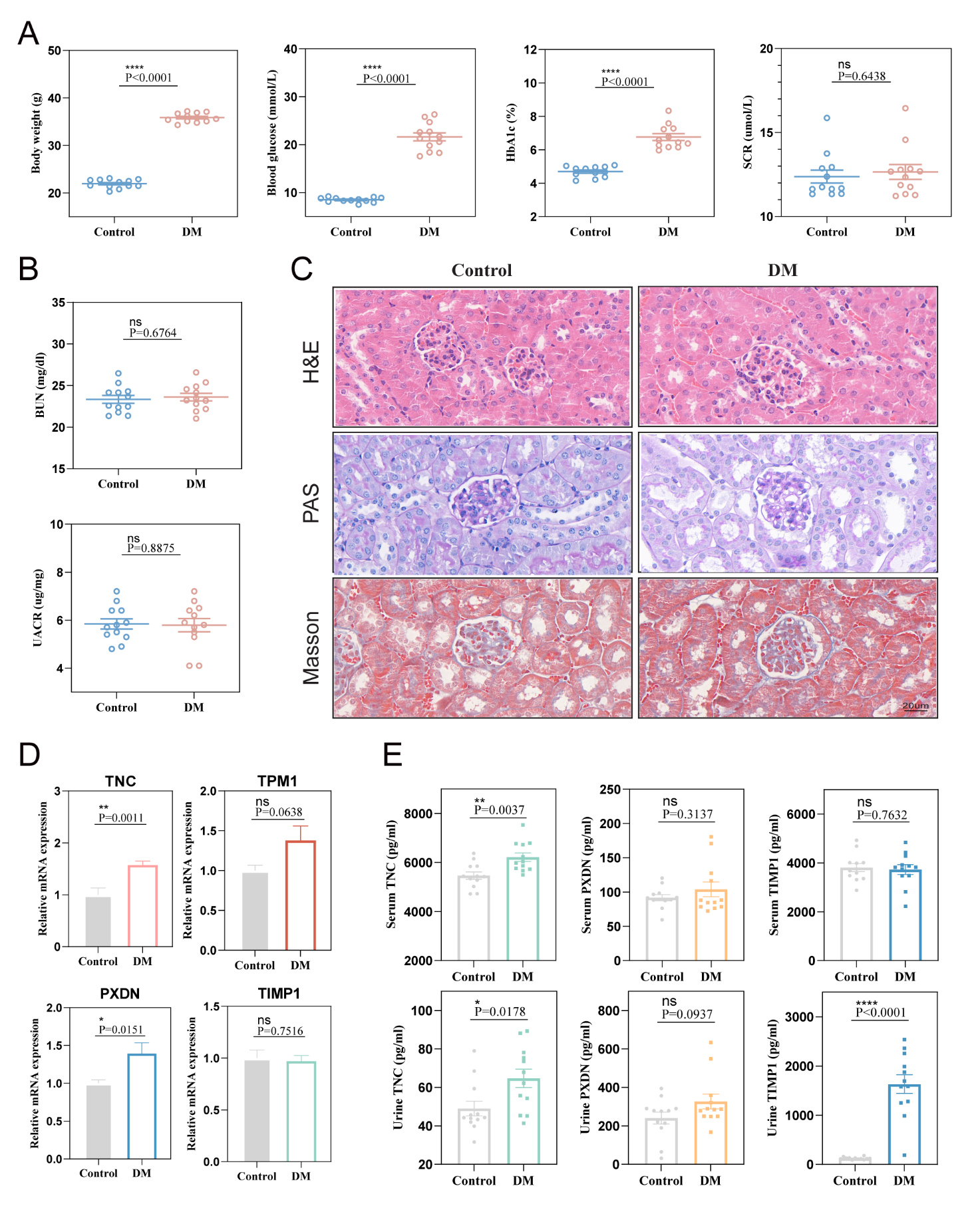


**Supplementary Figure 8.** Validation of diagnostic markers in a mouse model of diabetes. (A, B) The levels of body weight, blood glucose, HbA1c, serum creatinine, blood urea nitrogen, and urine albumin-creatinine ratio in mice. (C) Hematoxylin and eosin (H&E), periodic acid Schiff (PAS), Masson staining of mouse kidney. (D) mRNA expression levels of four diagnostic markers in kidney tissue. (E) Expression levels of markers in blood and urine.
